# Effects of antenatal dexamethasone treatment on glucocorticoid receptor and calcyon gene expression in the prefrontal cortex of neonatal and adult common marmoset monkeys

**DOI:** 10.1186/1744-9081-6-18

**Published:** 2010-03-22

**Authors:** Rochellys Diaz Heijtz, Eberhard Fuchs, Joram Feldon, Christopher R Pryce, Hans Forssberg

**Affiliations:** 1 Department of Neuroscience, Karolinska Institutet, S-171 77, Stockholm, Sweden; 2 Stockholm Brain Institute, Sweden; 3 Department of Women's and Children's Health, Astrid Lindgren Children's Hospital, Karolinska Institutet, S-171 776, Stockholm, Sweden; 4 Clinical Neurobiology Laboratory, German Primate Center, Göttingen, Germany; 5 Behavioural Neurobiology Laboratory, Swiss Federal Institute of Technology-Zurich, CH-8603 Schwerzenbach, Switzerland; 6 Clinic for Affective Disorders and General Psychiatry, Psychiatric University Hospital Zurich, CH-8008 Zurich, Switzerland

## Abstract

**Background:**

Synthetic glucocorticoids such as dexamethasone (DEX) are commonly used to promote fetal lung maturation in at-risk preterm births, but there is emerging evidence of subsequent neurobehavioral abnormalities in these children e.g. problems with inattention/hyperactivity. However, molecular pathways mediating effects of glucocorticoid overexposure on motor and cognitive development are poorly understood.

**Methods:**

In this study with common marmoset monkeys, we investigated for neonatal and adulthood effects of antenatal DEX treatment on the expression of the corticosteroid receptors and also calcyon, a risk gene for attention-deficit/hyperactivity disorder, in the prefrontal cortex (PFC). Pregnant marmosets were exposed to DEX (5 mg/kg body weight) or vehicle during early (days 42-48) or late (days 90-96) stages of the 144-day pregnancy.

**Results:**

In neonates, relative to controls, glucocorticoid receptor (GR) mRNA levels were significantly reduced after the late DEX treatment in the medial, orbital and dorsal PFC and after the early DEX treatment in the dorsal PFC. The early DEX exposure, specifically, resulted in significant reduction in calcyon mRNA expression in the medial, orbital, dorsal and lateral PFC relative to controls. Mineralocorticoid receptor (MR) mRNA levels were not significantly affected by DEX treatment. In adults, PFC GR, calcyon, and MR mRNA levels were not significantly affected by early or late prenatal DEX treatment.

**Conclusion:**

These findings indicate that antenatal DEX treatment could lead to short-term alterations in PFC expression of the GR and calcyon genes, with possible neurodevelopmental functional consequences.

## Background

Epidemiological studies indicate that children exposed to elevated levels of glucocorticoids during fetal life because of maternal stress during pregnancy have an increased risk of developing hyperactivity and cognitive deficits, e.g., attention-deficit/hyperactivity disorder (ADHD) [1,2]. In spite of this potential risk, synthetic glucocorticoids such as dexamethasone (DEX) and betamethasone are widely used during pregnancy to prevent respiratory distress syndrome in preterm infants. The pulmonary benefits are undisputed, but there is growing concern that the use of repeated courses of glucocorticoids prenatally could disturb normal brain development [3]. This is because the human fetal brain expresses glucocorticoid receptors (GR) and synthetic glucocorticoids could therefore target the developing brain and contribute to behavioural disturbances [4]. This hypothesis is consistent with a study conducted in school-aged children who received three or more courses of antenatal betamethasone, which found that such treatments are associated with an increased risk of postnatal aggressive/destructive behaviour, increased distractibility, and hyperactivity [5]. Recently, we demonstrated that prenatal exposure to DEX in a primate, the common marmoset, had an impact on social development, skilled motor reaching/dexterity, motivational and learning functions [6]. Overall, these findings suggest that prenatal DEX leads to altered development and functioning of critical brain circuits involved in cognition and behaviour.

The molecular pathways that could mediate the causal relationship between prenatal stress or glucocorticoid overexposure and development of neurobehavioral problems in children/adolescents remain poorly understood. Several susceptibility alleles have been shown to be associated with the risk of developing ADHD [7], and other psychiatric disorders [8], where motor and cognitive functions are often disrupted. There is only very limited information about whether prenatal glucocorticoid overexposure affects the expression of these risk genes. Recently, gene linkage and association studies have implicated the region of chromosome 10q, which contains the calcyon locus, with both the hyperactive/impulsive and inattentive symptoms of ADHD [9,10]. This association is supported by animal studies (e.g. using genetically engineered mice) showing the involvement of calcyon in the regulation of behavioral functions which are compromised in ADHD [11-13]. Anatomical studies have demonstrated that calcyon is highly expressed in multiple brain regions, including the prefrontal cortex (PFC), in both non-human primates and rodents [14,15]. The PFC is involved in mediating numerous cognitive-executive functions, e.g. working memory, decision-making, inhibitory response control, and attentional set-shifting, of central relevance to ADHD [16].

Within the framework of a multi-centre in vivo-ex vivo study of the effects of prenatal DEX exposure on offspring development in a nonhuman primate (Glucocorticoid Hormone Programming in Early Life and Its Impact on Adult Health, EUPEAH), the present study was conducted in the common marmoset (*Callithrix jacchus*) to investigate the effects of prenatal DEX on gene expression in the PFC. Two developmental stages were studied, namely neonate and adult. The genes studied were GR, the non-DEX sensitive mineralocorticoid receptor (MR), and calcyon. DEX was administered during early (days 42-48; late first third) or late (days 90-96; late second third) stages of the 144 day pregnancy. The early DEX exposure is likely to target the maturational stage of maximal neurogenesis in this primate [17], and therefore a putative sensitive period for inducing acute central effects of prenatal glucocorticoid overexposure (e.g. under maternal stress) with long-term consequences. The late DEX exposure is within the time-window when synthetic glucocorticoids are administered to pregnant women at risk of preterm delivery, and therefore of particular translational relevance. In humans, the accepted treatment schedules for a complete single course of antenatal corticosteroids are either two injections of betamethasone (12 mg) 24 hours apart, or four injections of DEX (6 mg) 12 hours apart [18]. Therefore, the regimen schedule of DEX used in the present study might be relevant to the use of multiple courses of antenatal corticosteroids in clinical practice.

## Methods

### Subjects

The common marmosets studied were bred at the Laboratory for Behavioural Neurobiology, Swiss Federal Institute of Technology Zurich, as described previously [6,19]. While twinning is the norm in this species, more than 50% of breeding females have litters of 3 neonates in captivity. Natural rearing of all three triplets is generally not possible and without intervention the weakest young dies some days after birth. Therefore, in triplet litters one neonate was euthanized on postnatal day 2 and these subjects were used to investigate the effects of prenatal DEX at the neonatal stage. For the present study, there were three neonates per treatment group, with each neonate derived from a different breeding pair. The sex of the neonates was: VEH: 3 males; EDEX: 3 males; LDEX: 1 female, 2 males. The surviving twin offspring per birth were used to investigate the long-term effects of DEX on physical growth, hypothalamic-pituitary-adrenal (HPA) axis, social behaviour, and neuropsychological function, from infancy to young adulthood, as described previously [6,19]. These subjects were euthanized at age 18-20 months and brains and other organs were collected. For the present study there were 6 VEH adults (3 female, 3 male), 6 EDEX adults (3 female, 3 male) and 6 LDEX adults (3 female, 3 male). The *in vivo *studies were conducted under experimental permit in accordance with the Swiss Animal Protection Act (1978). The brain tissues were shipped to Stockholm for study under license from the Convention for International Trade in Endangered Species of Wild Fauna and Flora (CITES), administered by the Swiss Federal Office for Veterinary Affairs and the Swedish Department of Agriculture.

### Prenatal DEX treatment

The common marmoset has a fully functional hypothalamic-pituitary-adrenal (HPA) axis, and exhibits relatively high physiological basal cortisol concentrations [20]. To determine a suitable treatment dose of DEX, a pilot study was conducted at the German Primate Center in which pregnant marmosets were treated with a range of doses of DEX, between 0.05 mg/kg and 10 mg/kg per day orally. Dexamethasone (0.5, 1.5 or 4 mg, Jenapharm^®^, Jena, Germany) was dissolved in 0.4 mL tap water and mixed with 1.6 mL NutiCal^® ^(Albrecht, Aulendorf, Germany). The mixture was taken voluntarily by all animals. 1 mg/kg or 5 mg/kg DEX per day was found to effectively suppress maternal endogenous cortisol production without adverse effects, whilst 10 mg/kg induced abortion and caused symptoms of glucocorticoid deficiency after cessation of treatment. The 5 mg/kg dose of DEX was used in all subsequent experiments and administered at the same time of day (09:00 h) to all pregnant females in the study. This dose is significantly higher than those shown to produce programming effects in rodents [21] or vervet monkeys [22], and reflects the relative resistance of the marmoset to glucocorticoid action [20]. Briefly, pregnant marmosets (n = 4 females per group) were allocated randomly to one of the treatment groups and were administered DEX (5 mg/kg) or syrup vehicle, during estimated gestation days 42-48 (early DEX, EDEX) and 90-96 (late DEX, LDEX) of the 144-day gestation period, as follows: vehicle (VEH at both treatment windows), early DEX (EDEX, and VEH late), late DEX (VEH early and LDEX), as described previously [6,19].

### Tissue collection and in situ hybridization

At 1400 h, a subject was removed from its home cage to a procedures room and sedated; deep anaesthesia was followed by rapidly removing the brain from the skull and freezing in isopentane on dry ice at -40°C, and storing at -80°C. Brains were coronally sectioned in a cryostat at 20 *μ*m thickness and prepared for *in situ *hybridization technique (ISH). For GR and MR, we used common marmoset-specific riboprobes that have been previously described [23]. For calcyon, a new riboprobe was prepared by amplifying a conserved region of calcyon (GenBank: AF225903, 301-660) from a human brain cDNA library. The amplified cDNA fragment was subcloned into a pCR1II-TOPO vector (Invitrogen, Lidingö, Sweden), and confirmed by nucleotide sequencing. Linearized plasmids were used to synthesize [^35^S] UTP-labeled riboprobes. *In-vitro *transcription was carried out using the MAXIscript™ SP6/T7 kit (Applied Biosystems, Uppsala, Sweden) and [α35-S]UTP (NEG039H; Perkin Elmer, Upplands Väsby, Sweden) according to the manufacturer's instructions. The transcripts were purified using NucAway™ spin columns (Applied Biosystems, Uppsala, Sweden). Fixation, pre-hybridization, hybridization and washes were performed as previously described [12].

*In situ *hybridization was conducted with sections from the intermediate PFC, using two adjacent sections per subject. For each probe, all sections for the same age group were included in a single ISH run. Controls comprised concurrent hybridization with sense strand probes. The sections were exposed to autoradiographic film (BIOMAX, Kodak) for 1-2 weeks to enable visualization of GR-, MR- and calcyon-hybridization. Calibrated [^14^C]-labelled standards (Amersham Biosciences, Uppsala, Sweden) were incorporated in all cassettes to allow for quantification.

### Image and data analysis

Films were scanned with an Epson Perfection 4990 scanner as grey scale film, using 800 pixels and saved as high-quality JPEGs. Optical density values were quantified using appropriate software (NIH Image J version 1.29, U.S. National Institutes of Health, Bethesda, MD, USA). The following PFC regions were identified according to [24]: medial, orbital, dorsal and lateral. In each region, signals were measured over the two adjacent sections per subject and the mean value per subject was used for statistical analysis. For each gene, the effects of prenatal DEX treatment were first studied in a mixed-model ANOVA that included developmental stage (neonate, adult) as a between-subjects factor. ANOVA was then performed for each developmental stage separately using a two-stage approach: Firstly, a mixed ANOVA with PFC subregion and hemisphere as within-subjects factor, and treatment (VEH, EDEX and LDEX) as between-subject factor. Where mixed ANOVA revealed a main effect of treatment or a treatment × subregion interaction, treatment differences were examined in each subfield separately using univariate ANOVA. Significant main effects were followed up *post hoc *using the Bonferroni/Dunn test. All but one neonate was male. Sex was included as a factor in the adult ANOVA and as there were no significant effects of sex, sex is not reported on as a factor in the Results. For all analyses, significance was assigned at the P < 0.05 level. All data are presented as means ± S.E.M.

## Results

### Effects of prenatal DEX on anthropometric parameters in neonates

As found in earlier studies [19], in two-day-old monkeys there were no significant effects of prenatal DEX on body weight (g), (VEH: 27.6 ± 1.3, EDEX: 27.8 ± 0.7, LDEX: 26.1 ± 1.3; P > 0.1) or knee-heel length (mm) (VEH: 26 ± 0, EDEX: 26 ± 0.6, LDEX: 26 ± 0.6; P > 0.1).

### Effects of prenatal DEX on calcyon, GR and MR mRNA expression

**Figure 1:**
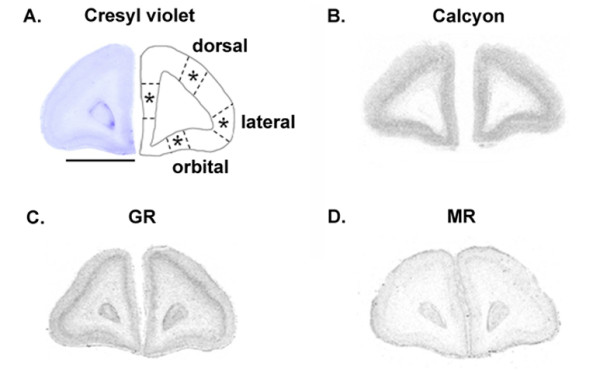
**Representative autoradiograms showing calcyon, GR and MR mRNAs in PFC of neonate marmoset monkeys. A**. Low-power scan of a cresyl violet-stained coronal section taken from an intermediate level of the marmoset frontal lobes (left). The approximate boundaries used to delineate the medial, dorsal, lateral and orbital PFC subregions are indicated with dotted lines (right). **B**. Calcyon mRNA expression. **C**. GR mRNA expression. **D**. MR mRNA expression. Scale bar = 4 mm.

**Figure 2:**
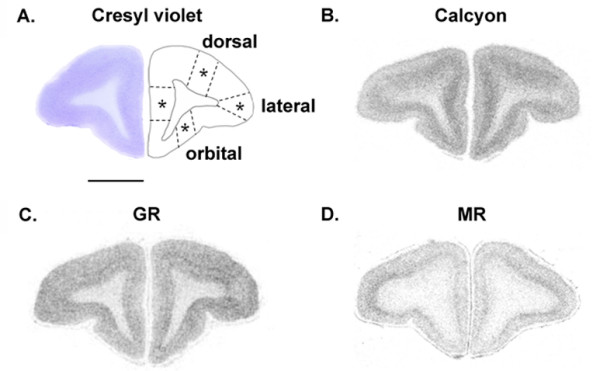
**Representative autoradiograms showing calcyon, GR and MR mRNAs expression in PFC of adult marmoset monkeys. A**. Low-power scan of a cresyl violet-stained coronal section taken from an intermediate level of the marmoset frontal lobes (left). The approximate boundaries used to delineate the medial, dorsal, lateral and orbital PFC subregions are indicated with dotted lines (right). **B**. Calcyon mRNA expression. **C**. GR mRNA expression. **D**. MR mRNA expression. Scale bar = 4 mm.

Each transcript was detected (Figs. 1, 2) in both neonate and adult PFC. The specificity of signal was confirmed using sense probes, which did not produce any signals (see Additional file 1). It is worth mentioning that we also tested the specificity of the calcyon probe using human brain tissue: here also the sense probe produced negligible signals (R. Diaz Heijtz and T. Hökfelt, unpublished data). Hemisphere was included as a factor in the analyses and as there were no significant effects of hemispheres, hemisphere is not reported as a factor in the Results. In all genes studied, the ANOVA of age group (neonate, adult) × treatment (VEH, EDEX, LDEX) × PFC subregion yielded a significant main effect of age (P < 0.0001) and no significant effect of treatment. A *posteriori *ANOVAs were then performed separately for neonates and adults, reported below.

#### Neonatal PFC

**Figure 3:**
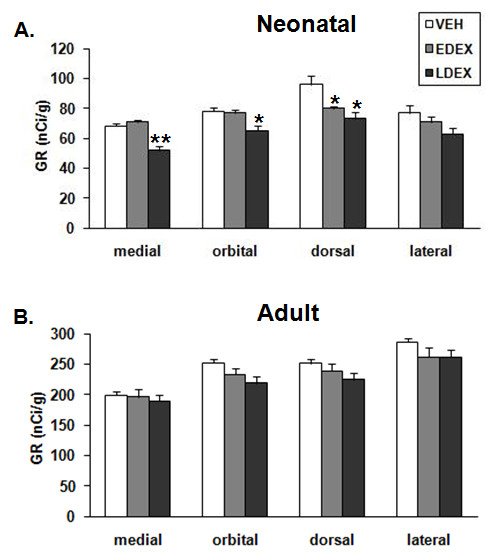
**Prenatal DEX effects on the expression of GR mRNA in PFC**. PFC subregion-specific densities are presented (mean ± S.E.M.) for neonate (A) and adult (B) monkeys. **P < 0.001, * P < 0.05 when compared to VEH of the same PFC region.

**Figure 4:**
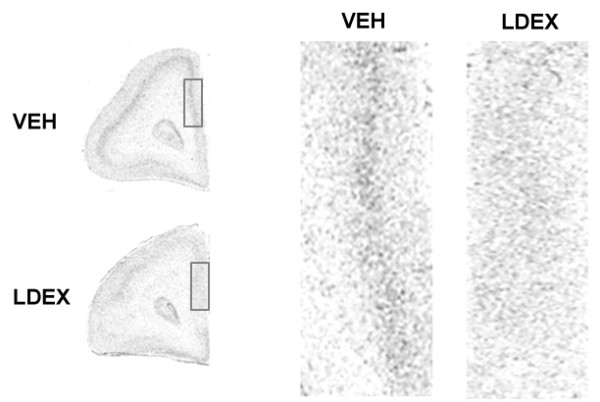
**Representative autoradiograms showing the effects of LDEX treatment on GR mRNA expression in PFC of neonates**. Abbreviation as follows: VEH, vehicle; LDEX, late dexamethasone exposure. For more details see Fig. 1.

For GR mRNA, mixed ANOVA revealed a significant treatment-by-PFC subregion interaction (F (6, 45) = 2.5, P = 0.0375), and significant main effects of treatment (F (2, 15) = 14.5, P = 0.0003) and PFC subregion (F (3, 45) = 23.1, P < 0.0001). Subsequent analysis showed that GR mRNA (Figs. 3A and 4) was significantly affected by prenatal DEX treatment in the medial (F(2,15) = 28.3, P < 0.001), orbital (F(2,15) = 8.2, P = 0.004), and dorsal (F(2,15) = 7.7, P = 0.005) PFC. Post-hoc analysis showed that LDEX neonates expressed significantly (P < 0.05) lower levels of GR mRNA in the medial and orbital PFC when compared to VEH neonates. In the dorsal PFC both EDEX and LDEX neonates expressed significantly (P < 0.05) lower levels of GR mRNA than the VEH neonates.

In the case of MR mRNA there were no significant main or interaction effects (P > 0.05) (Table 1).

**Table 1:** MR mRNA levels (nCi/g, mean ± S.E.M.) in VEH, EDEX and LDEX Marmosets

Prefrontal cortex subregions				
	**medial**	**orbital**	**dorsal**	**lateral**
**Neonates**				
VEH	58 ± 2.6	62 ± 4.4	65 ± 6.5	60 ± 6.9
EDEX	56 ± 1.1	54 ± 3.5	54 ± 2.4	51 ± 3.7
LDEX	61 ± 3.0	65 ± 4.3	75 ± 5.9	68 ± 5.6
**Adults**				
VEH	166 ± 5.3	171 ± 5.4	226 ± 5.8	242 ± 7.4
EDEX	168 ± 12.9	173 ± 10.0	221 ± 17.2	229 ± 18.0
LDEX	189 ± 7.3	193 ± 7.3	244 ± 16.0	266 ± 25.5

For logistic reasons, analysis of prenatal DEX effects on prefrontal cortex mineralocorticoid receptor (MR) was conducted with matched sections from only 4-6 subjects/manipulation group. There were no significant main or interaction effects of DEX (P > 0.05).

**Figure 5:**
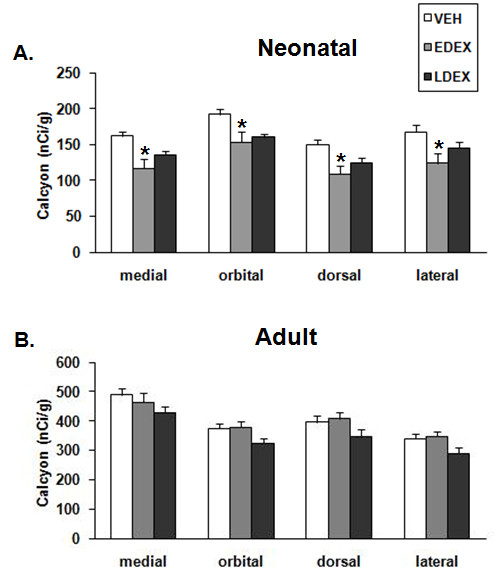
**Prenatal DEX effects on the expression of calcyon mRNA in PFC**. PFC subregion-specific densities are presented (mean ± S.E.M.) for neonate (A) and adult (B) monkeys. * P < 0.05 when compared to VEH of the same PFC region.

**Figure 6:**
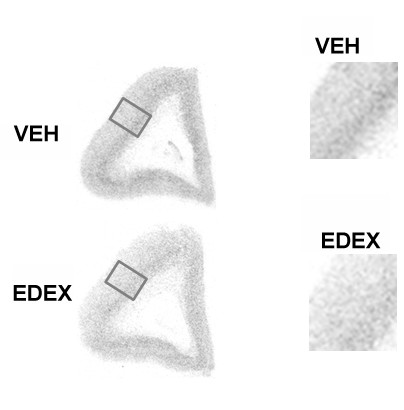
**Representative autoradiograms showing the effects of EDEX treatment on calcyon mRNA expression in PFC of neonates**. Abbreviation as follows: VEH, vehicle; EDEX, early dexamethasone exposure. For more details see Fig. 1.

For calcyon mRNA, mixed ANOVA revealed significant main effects of treatment (F (2, 15) = 6.1, P = 0.0116) and PFC subregion (F (3, 45) = 37.8, P < 0.0001). Subsequent analysis showed that calcyon mRNA (Figs. 5A and 6) was significantly affected by prenatal DEX treatment in the medial (F(2,15) = 7.1, P = 0.007), orbital (F(2,15) = 4.2, P = 0.035), dorsal (F(2,15) = 5.5, P = 0.016), and lateral (F(2,15) = 4.0, P = 0.041) PFC. Post-hoc analysis with Bonferroni/Dunn test showed that in the medial, orbital, dorsal and lateral PFC, EDEX neonates expressed significantly (P < 0.05) lower levels of calcyon mRNA compared to VEH neonates. The mean decrease in calcyon mRNA expression in LDEX neonates was not significant.

#### Adult PFC

For GR, MR and calcyon mRNAs, there was no siginificant effect of treatment in adult subjects. For GR (Fig. 3B), repeated ANOVA revealed significant PFC subregional differences in mRNA expression (F (3,99) = 87.9, P < 0.0001); with the lPFC and mPFC expressing the highest and lowest mRNA levels, respectively. For MR (Table 1), repeated ANOVA revealed significant PFC subregional differences in expression (F (3, 81) = 84.5, P < 0.0001); with the lPFC and dPFC subregions expressing the highest mRNA levels. In the case of calcyon (Fig. 5B), repeated ANOVA revealed significant PFC subregional differences in mRNA expression (F (3, 99) = 100.9, P < 0.001), with the mPFC subregion expressing the highest mRNA levels.

## Discussion

This study describes the effects of prenatal DEX treatment on gene expression in the PFC, a brain region implicated in ADHD and other psychiatric disorders, at neonate and adult life stages. Thus, prenatal DEX exposure in marmoset monkeys leads to alterations in GR and calcyon (a risk gene for ADHD) mRNA expression in the neonate PFC. Taken together with recent findings (see below), the present results suggest that in non-human primates antenatal DEX exposure might modify the early postnatal development and synaptic plasticity of PFC with possible functional consequences.

Studies in rodents have shown that the developing PFC is extremely sensitive to stress, possibly including elevated levels of stress hormones (i.e. glucocorticoids). For example, prenatal stress in rodents leads to lateralized changes in PFC DA function [25,26], alterations of PFC neuronal structure (e.g. spine density and dendritic complexity; [27]), and long-lasting changes in some subtypes of dopamine and glutamate receptors [27], which are thought to underlie cognitive and emotional deficits observed in prenatally stressed animals [26]. In humans, maternal stress during pregnancy is associated with an increased risk of developing ADHD-like symptoms [1,2,26]. Moreover, antenatal exposure to multiple courses of synthetic glucocorticoids is also associated with increased neurobehavioral problems (e.g. aggressive/destructive behaviour, increased distractibility, and hyperactivity) in children [5,28,29]. Taken together, these results suggest that fetal glucocorticoid overexposure leads to altered development and functioning of PFC, a critical region involved in cognition and emotional regulation.

Recently, we described the effects of prenatal DEX exposure on behavioral traits in juvenile and adolescent common marmosets. We showed for the first time that exposure of the fetus to DEX has a long-term postnatal impact on social development, skilled motor reaching/dexterity, motivational and learning functions [6]. In the present study, we assessed whether the same treatment could affect expression of corticosteroid receptors and calcyon (a risk gene for ADHD; see below) in neonate and adult PFC. Prenatal DEX exposure, especially during late gestation, led to significant reductions in GR expression in the neonate PFC. The expression of MR mRNA was not significantly affected by prenatal DEX treatment, which is consistent with the fact that DEX has a low affinity for the MR and selectively binds to GR [30]. A recent study showed that in the developing human hippocampus both GR and MR are expressed between 24 and 34 weeks of gestation [31]. This is the time-window when synthetic glucocorticoids are administered to pregnant women at risk for preterm delivery. Interestingly, the same study did not find an effect of antenatal DEX treatment on GR or MR gene expression in the human hippocampus at the third trimester of gestation. Based upon previous work in the squirrel monkey [32] and rhesus monkey [33] demonstrating the relatively low expression of GR in hippocampus versus neocortex, and the present results, we postulate that, in primates, GR in neocortical areas are more important potential mediators of effects of antenatal glucocorticoid treatment than are GR in hippocampus.

One of the most interesting findings of the present study is the observation that prenatal DEX treatment induced a down-regulation of calcyon (a risk gene for ADHD) gene expression in the neonate PFC. Calcyon is a single transmembrane protein highly expressed in PFC [12,14]. New evidence indicates a role for calcyon in clathrin mediated endocytosis, a critical component of synaptic plasticity [34]. This is consistent with anatomical findings localizing calcyon to vesicular compartments in dendritic spines and axon terminals [35], two sites in the brain where clathrin mediated endocytosis is essential for efficient neurotransmission and plasticity associated with learning and memory [34]. Support for a role of calcyon in the aetiology of ADHD comes primarily from genetic studies. In a recent genome-wide linkage study for loci influencing ADHD, the calcyon gene was found to coincide with one of the highest positive linkage sites identified at chromosome 10q26 [9]. More recently, the inheritance of nine polymorphisms in the calcyon gene was examined with ADHD and their immediate families using the transmission disequilibrium test [10]. This study reported evidence for excess transmission of the most common calcyon haplotype, designated C1. In addition, C1 was positively associated with both hyperactive/impulsive and inattentive symptoms, supporting the idea that variations in calcyon may contribute to both deficits in motor control and cognitive functions of the disorder. This notion is supported by recent animal studies demonstrating alterations in calcyon gene expression in a genetic rat model of ADHD [12] and ADHD-like phenotypes in calcyon over-expressing transgenic mice [13]. In the current study we showed that calcyon is expressed during early postnatal development of the non-human primate PFC and that it is sensitive to fetal glucocorticoid overexposure. These observations have marked translational significance given the genetic link of calcyon to ADHD.

DEX is known to cause its effects by binding to the GR [30]. Once activated, the GR subunits homodimerize and bind to DNA via glucocorticoid responsive elements (GREs) in the promoter region of target genes resulting in the regulation of gene expression [36]. In addition, GR is also involved in crosstalk with other transcription factors, such as AP-1, CREB-binding protein, STAT5, and NFkB (see [37]). Crosstalk between GR and these transcription factors may occur either through composite response element, overlapping response element, or interaction between GR and transcription factors. We used bioinformatic tools (MatInspector, Genomatix Software GmbH) to search for the GRE consensus sequence (consisting of the palindromic binding site AGAACAnnnTGTTCT) and related elements in the human CALY promoter. This analysis revealed that the human CALY promoter does not contain a consensus sequence for the GRE (see Additional file 2), suggesting that antenatal DEX-mediated decreases in calcyon gene expression are not likely to be a direct effect of GR binding to the calcyon promoter. However, the CALY promoter contains consensus sequences for the binding of several transcription factors including Sp-1 (see Additional file 2). Interestingly, previous studies have shown that DEX can regulate gene transcription through a mechanism dependent on the Sp-1 transcription factor [38], and therefore could be a potential mechanism mediating antenatal DEX effects on calcyon gene expression.

In our previous studies in marmoset monkeys, we observed that the outcome of antenatal DEX exposure on behavioral traits was dependent upon the timing of administration i.e. EDEX versus LDEX [6,19]. In the present study, we also observed that early and late DEX treatments differentially affected GR and calcyon mRNA levels in neonates, with early DEX affecting calcyon and late DEX affecting GR. We hypothesize that the different effects of early vs. late DEX treatment on gene expression and behavior could originate from the difference in the stage of brain maturation (e.g. GR expression and/or transcription factors interaction with GR) at the time of these two antenatal DEX treatments.

The absence of significant effects of antenatal DEX on either GR or calcyon gene expression in adult PFC suggests the presence of compensatory - and possibly but not necessarily protective - mechanisms acting during the protracted postnatal maturation of the primate PFC [39,40]. However, it will be important to assess in future studies potential effects of repeated antenatal glucocorticoid exposure on PFC gene expression and cognitive-executive functions during the juvenile period when ADHD-symptoms usually manifest in children.

## Conclusion

In summary, in the present study we investigated the effects of antenatal DEX treatment on the expression of corticosteroid receptors and calcyon, a risk gene for ADHD, in the PFC in the common marmoset. Our results indicate that prenatal DEX exposure led to alterations in neonate PFC gene expression of GR and calcyon, a risk gene for ADHD. These novel primate findings suggest a potential molecular pathway mediating the effects of fetal glucocorticoid overexposure on abnormal motor and cognitive development.

## List of abbreviations

ADHD: attention-deficit/hyperactivity disorder; DEX: dexamethasone; GR: glucocorticoid receptor; MR: mineralocorticoid; PFC: prefrontal cortex.

## Competing interests

The authors declare that they have no competing interests.

## Authors' contributions

RDH designed and carried out the study, conducted statistical analyses, and drafted the manuscript. CRP carried out the in vivo study. EF, JF, CRP and HF participated in the overall study design and helped draft the manuscript. All authors read and approved the final manuscript.

## Supplementary Material

Additional file 1 **calcyon sense RNA probe**. Representative autoradiogram showing a PFC section of neonate marmoset monkey hybridized with calcyon sense RNA probe. Click here for file

Additional file 2 **Transcription binding site analysis of the human calcyon promoter**. This table shows potential transcription binding sites in the human calcyon promoter as predicted by the MatInspector analysis (Genomatix Software GmbH). Click here for file
