# Supplementary figures and images for: Effects of antenatal dexamethasone treatment on glucocorticoid receptor and calcyon gene expression in the prefrontal cortex of neonatal and adult common marmoset monkeys
Source: Behav Brain Funct. 2010 Mar 22;6:18. doi: 10.1186/1744-9081-6-18 (PMC2858712; doi:10.1186/1744-9081-6-18)

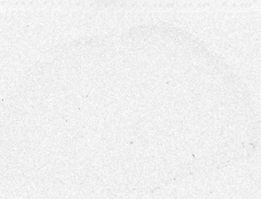

Supplement: Additional file 1 — calcyon sense RNA probe. Representative autoradiogram showing a PFC section of neonate marmoset monkey hybridized with calcyon sense RNA probe. [file 1744-9081-6-18-S1.JPEG]
